# Impact of IDO1 and IDO2 on the B Cell Immune Response

**DOI:** 10.3389/fimmu.2022.886225

**Published:** 2022-04-13

**Authors:** Lauren M. F. Merlo, Weidan Peng, Laura Mandik-Nayak

**Affiliations:** Lankenau Institute for Medical Research, Wynnewood, PA, United States

**Keywords:** IDO1, IDO2, B cells, autoimmunity, inflammation

## Abstract

Indoleamine-2,3-dioxygenase (IDO)1 and IDO2 are closely related tryptophan catabolizing enzymes that have immunomodulatory properties. Although initially studied as modifiers of T cell activity, emerging evidence suggests IDO1 and IDO2 also have important roles as modulators of B cell function. In this context, IDO1 and IDO2 appear to play opposite roles, with IDO1 inhibiting and IDO2 driving inflammatory B cell responses. In this mini review, we discuss the evidence for IDO1 and IDO2 modulation of B cell function, focusing on the effect of these enzymes on autoimmunity, allergic responses, protective immunity, and response to pathogens. We summarize strategies to target IDO1 and/or IDO2 as potential therapeutics for inflammatory autoimmune disease and highlight outstanding questions and areas that require future study.

## Introduction

The indoleamine 2,3-dioxygenase proteins (IDO1 and IDO2) are a pair of enzymes resulting from an ancient gene duplication (1, 2) that can catabolize the amino acid tryptophan (a 3^rd^ tryptophan catabolizing enzyme, TDO, is evolutionarily unrelated). In addition to this direct connection to amino acid metabolism, IDO1 and IDO2 also have a variety of functions related to immunity, with the two proteins playing a role in multiple immune cell types. Historically, the IDO pathway was first linked to immune regulation by Munn and Mellor (3), who identified an important role for IDO1 in maternal-fetal tolerance. Consistent with this function in promoting immune tolerance, later work demonstrated IDO1 also contributes to tumor immune evasion, acting in antigen presenting cells (APCs) to promote T cell tolerance (4). IDO2 was discovered more recently, and appears to act differently than IDO1, promoting rather than repressing inflammation, particularly in certain autoimmune contexts. Because IDO1 and IDO2 may have opposing functions, it is possible to conflate the roles of the two enzymes, particularly with the use of nonspecific inhibitors such as 1-methyltryptophan (1MT). To avoid any confusion and to clarify the distinct roles of IDO1 and IDO2, this review will focus on studies using knockout models (predominantly restricted to the C57BL/6 background) and, where possible, specific inhibitors of individual enzymes (amenable to models on different genetic backgrounds).

Much of the previous work relating IDO function to immunity has centered on the association between IDO1 and regulatory T cells, but there has been a growing body of work suggesting that IDO1 and IDO2 also play a critical role in B cells. Both IDO1 and IDO2 are expressed in B cells but the contribution of each to promote or inhibit B cell tolerance or inflammation is less well established. Here, we review the emerging evidence pointing to the importance of the IDO pathway in B cell-mediated immune responses, discuss the potential for therapeutic targeting of the IDO enzymes, and highlight areas warranting future study.

## IDO1 and IDO2 in B Cell Development and Differentiation

IDO1 is expressed in a variety of tissue types including colon, lung, lymph nodes, placenta, testis, epididymis, thyroid, and spleen (5, 6). IDO2 is expressed at a lower level and in a more restricted set of tissues, including liver, kidney, lymph nodes, and placenta (5, 7, 8). Both IDO1 and IDO2 mRNA are expressed within the B cell compartment, particularly in response to activation. Although IDO1 and IDO2 expression is low in naïve B cells, both enzymes are strongly upregulated in response to stimuli. IDO1 in B cells increases with exposure to T-independent type I antigens, Toll-like receptor agonists LPS and CpG, and B cell receptor (BCR) crosslinking with anti-IgM, but not with the T cell help mimic CD40L (9). IFNγ exposure dramatically increases IDO1 expression (e.g. (10). IDO2 mRNA is also upregulated by LPS, CpG, and CD40L but not BCR crosslinking alone. Upregulation in IDO2 was much stronger when paired with the cytokines IL-4 or IL-21, but unlike IDO1, IFNγ did not have a significant effect on IDO2 expression (11).

Naïve mice lacking either IDO1 or IDO2 show no systematic changes in immune cell profiles, with normal development and numbers of B cells in the bone marrow and periphery. Likewise, IDO1 and IDO2 deficient mice had normal differentiation into peripheral B cell subsets, including transitional, follicular, and marginal zone B cells (9, 12). Serum Ig levels are similar in wild-type vs knockout mice, suggesting that while IDO may affect disease-specific antibody responses, it does not affect total antibody production (13). Importantly, IDO1 and IDO2 are not redundant or compensatory, as double knockout mice lacking both IDO1 and IDO2 show no differences in B cell development or antigen presentation (14).

## Effect of IDO1/IDO2 in B Cells on Autoimmunity

The clearest evidence for the role of the IDO pathway in B cells comes from studying small animal models of autoimmune disease. Both IDO1 and IDO2 affect B cell-mediated models of autoimmune arthritis, though the roles of IDO1 and IDO2 are clearly distinct. Although alterations in serum and urine levels of the tryptophan metabolite kynurenine have long been associated with disease (e.g. (15, 16), the specific contributions of IDO1 and IDO2 were first implicated in studies using the well-established KRN T cell receptor transgenic mouse model of arthritis (17) and the general IDO inhibitor 1MT (18). To parse the specific roles of the two IDO enzymes, subsequent experiments using genetic knockouts yielded the surprising result that deletion of IDO1 does not affect disease, whereas deletion of IDO2 causes an amelioration in arthritis (13). This reduction in arthritis was associated with a decrease in autoantibody secreting cells in IDO2 ko mice. Confirming that this effect wasn’t due to alterations in the expression of IDO1 following deletion of IDO2, double knockout mice lacking *both* IDO1 and IDO2 show the same reduction in arthritis as is seen in IDO2 knockouts alone (14). This demonstrates that IDO1 and IDO2 have distinct roles in this system and that IDO2, rather than suppressing inflammatory responses, in fact promotes the development of autoimmune inflammation. Importantly, this proinflammatory effect of IDO2 on arthritis development was attributed specifically to its action in B cells. Using a series of adoptive transfer experiments, IDO2 in B cells was shown to be both necessary and sufficient for arthritis development. This response required B cells that were cognate and antigen specific, and IDO2 appears to be particularly potent in the marginal zone B cell compartment (19).

IDO has also been studied in other models of autoimmune arthritis, including models of juvenile idiopathic arthritis (JIA) and collagen-induced arthritis (CIA). IDO1 deletion was shown to have no effect on JIA (20); however, the effect of IDO2 deletion has yet to be tested in this model. Directly evaluating the role of IDO1 and IDO2 using genetically deficient mice is difficult in the CIA model due to reduced disease penetrance in the C57BL/6 strain (21). Although one study showed a slight exacerbation of disease in IDO1 ko C57BL/6 mice (22), most studies have relied on direct or indirect inhibitors in the DBA/1J mouse strain to assess the role of IDO function in the CIA model. Despite using the same DBA1/J strain, the effect of 1MT is inconsistent, with some groups showing an exacerbation of disease (22, 23) and others showing no effect (24, 25). In contrast to genetic deletion of IDO1, specific targeting of IDO1 *via* an adenovirus system generates a reduction of disease in a rat model of CIA (26). Targeting of IDO2 *via* an IDO2-specific antibody also results in amelioration of disease in the mouse CIA model (24). Like the KRN model, development of arthritis in the CIA model is mediated by autoantibodies, though the specific effect of IDO1 and IDO2 on the B cell compartment remains to be established. The differing roles of IDO1 and IDO2 in these different model systems of arthritis underscore the importance of using genetic knockouts or specific inhibitors of the individual IDO enzymes to properly assess IDO1 and IDO2 function.

The effect of the IDO pathway on autoimmunity extends beyond what has been documented in models of arthritis. Other B cell-mediated autoimmune and inflammatory diseases are also affected by IDO1 and IDO2, including multiple sclerosis (MS) and psoriasis. A common mouse model of MS, experimental autoimmune encephalomyelitis (EAE), has been studied with respect to both IDO1 and IDO2. EAE is exacerbated in mice genetically lacking IDO1 (27) but not IDO2 (28). The nonspecific inhibitor 1MT has been shown to exacerbate EAE in some studies (29, 30) but reduce disease in another (31). Of note, the opposing results were obtained using SJL vs. C57BL/6 genetic backgrounds. Using an imiquimod-induced mouse model of psoriasis, Fujii et al. (32) found that IDO1 did not influence disease, but that IDO2 may act to regulate inflammation in this system. Here, IDO2 ko mice were found to have enhanced disease, suggesting an anti-inflammatory role for IDO2 in this context, in contrast to the proinflammatory role of IDO2 in KRN arthritis. Although B cells play important roles in the pathogenesis of both MS and psoriasis, the potential role IDO1/2 plays in mediating B cell function in these models remains to be determined.

The role of IDO has also been examined in several models of systemic lupus erythematosus (SLE). SLE is driven by the production of antibodies against nuclear components, particularly dsDNA, but involves an intricate interplay between immune cell types in initiation and maintenance of disease. Because of the complex genetic background of many lupus models, it has been difficult to work with genetic knockouts of IDO1 and IDO2, though Davison et al. (33) found no differences in autoantibody production, immune cell activation pattern, or renal inflammation in B6.Nba2 mice lacking IDO1. Here, IDO1 expression was limited to plasmacytoid dendritic cells, which share some markers with B cells (CD19^+^), as well as macrophages. Additional studies with 1MT do suggest a role for IDO in the MRL/*lpr* model of lupus, though one study showed an exacerbation of disease (34) while another showed an amelioration of SLE (19).

## IDO and Allergic Responses

IDO1 and IDO2 have also been studied in a models of allergic inflammation. In the oxazolone-induced model of contact hypersensitivity (CHS), both IDO1 and IDO2 genetic knockouts have reduced CHS responses compared to wild-type controls (12). Although this is generally considered to be a model of T cell immunity, several studies have shown the importance of anti-hapten B cells, particularly B-1 B cells, and the associated antibody response to the pathogenic inflammatory response (35, 36). In a series of B cell add-back experiments similar to what was performed in the KRN arthritis model, addition of wild-type but not IDO2 ko B cells to the IDO2 genetic knockouts sensitized with oxazolone can restore a CHS response (11). This again supports the role of IDO2 in B cells in promoting inflammation. A second allergy-related model shows the importance of IDO1 in mediating immune responses. Xu et al. (37) demonstrate a reduction in Th2-related cytokines and IgE response in IDO1 ko mice in a model of allergic airway inflammation. The authors successfully identify a reduction in lung DCs as a component of this response, but the reduced IgE response suggests a potential role for B cells as well.

## Effect of IDO1/2 on Protective Immunity

The role of the IDO pathway on protective immunity is just beginning to be deciphered and has been evaluated both in terms of IDO1 and IDO2 in B cell responses to model antigens and pathogens. *In vivo* immunization experiments in wild-type and IDO1 ko mice using the T-dependent antigen NP-OVA, T-independent type I antigen NP-LPS, and T-independent type II antigen NP-Ficoll, showed significant increases in IgM response in the IDO1 ko mice to the two T-independent antigens but not to the T-dependent antigen. In the NP-Ficoll immunized mice, IgG_1_ and IgG_3_ were also increased (9). This suggests that IDO1 acts as a suppressor of B cell responses in this system, since antibody responses are exacerbated in the absence of IDO1. In contrast, Merlo et al. (14) found no differences in IgM responses to NP-Ficoll, NP-LPS, or the T-dependent antigen NP-KLH in IDO1 ko mice but did show a slight decrease in T-independent type II responses in IDO2 deficient mice, indicating that IDO2 may act to promote B cell responses.

The effect of IDO1 and IDO2 on immune responses to pathogens has been studied in several different systems. In influenza, elimination of IDO1 does not alter viral clearance or antibody production (14, 38), though other responses to flu infection, including influenza-specific CD8 T cell responses, may be mediated by IDO1 (38). In contrast, deletion of IDO2 results in a 50% reduction in antibodies, suggesting a direct effect of IDO2 on B cells in this system (14). IDO1 and IDO2 have also been examined in a model of endotoxin (LPS) shock, though the specific contribution of B cells was not directly examined. Here, loss of IDO1 has a protective effect and promotes host survival (39), potentially by restoring the balance between IL-10 and IL-12. Loss of IDO2 again has an opposing effect, with IDO2 ko mice demonstrating an increased inflammatory response and associated cytokine production, exacerbating disease (40). Finally, IDO1 has been proposed to provide a protective effect against pathogen-driven inflammation in aspergillosis, though this is thought to be mediated through IDO1 expression in DCs (41).

## Mechanism of Action

Studies on molecular and cellular mechanism of IDO1/2 support a direct connection between amino acid catabolism and immunity. Local depletion of tryptophan as mediated by IDO1 activates the amino acid sensing GCN2 kinase pathway. This, in turn, suppresses cell proliferation and leads to altered levels of immune-modulating cytokines such as IL-6 (42, 43). Deprivation of tryptophan by IDOs also inhibits the immunoregulatory kinases mTOR and PKC-Θ, along with a downstream effect in autophagy (44–46). In addition to the direct effect of Trp depletion, IDOs producing kynurenine and other tryptophan derivatives are natural immunologically active ligands for the aryl hydrocarbon receptor (AhR) (47). Activation of AhR by kynurenine upregulates IDO1 and IDO2 and promotes the generation of T regulatory cells that suppress adaptive immunity (48–50). However, Shinde et al. suggest that tryptophan metabolites do not alter B cell responses to some model antigens *in vitro* or *in vivo*, suggesting that GCN2 signals may be the dominant tryptophan-dependent molecular mechanism in B cells (9). It is also important to note that most of these studies look globally at tryptophan metabolism and do not distinguish separate effects of IDO1 and IDO2. Further studies will be needed to determine whether IDO1 and IDO2 affect immunity through the same pathway or through distinct mechanisms.

There is also evidence that IDO1/2 can regulate immunity through mechanisms independent of tryptophan catabolism. Pallotta et al. found IDO1 can act as an intracellular signaling molecule in plasmacytoid DCs, which share some characteristics with B cells. They propose that long-term maintenance of a regulatory phenotype occurs through a positive feedback loop involving TGFβ and the non-canonical NFκB pathway (51, 52). This non-enzymatic signaling function involves phosphorylation of IDO1 ITIM motifs by SH2-domain containing proteins such as SHP-1/SHP-2 and SOCS3 (reviewed in (53). Notably, IDO2 has only one ITIM motif (ITIM2) and is unlikely to share the mechanistic pathways identified for IDO1. There is, however, new evidence for a non-enzymatic role of IDO2 in B cells. A recent study from our group examined the effect of enzymatically inactive IDO2 on the development of arthritis and contact hypersensitivity (CHS) (54). While CHS is reduced in mice carrying the catalytically inactive IDO2, arthritis is unaffected. This suggests that enzymatic activity is critical for CHS development but does not play a role in arthritis development in this system. Importantly, B cell add-back experiments demonstrate the importance of this non-enzymatic role of IDO2 in B cells in arthritis, with enzymatically inactive IDO2-expressing B cells able to rescue arthritis in an otherwise IDO2 knockout system. The mechanism of action of this non-enzymatic role remains to be elucidated, but the study identified four diverse proteins with the potential to bind to IDO2 (54).

## Targeting Approaches

Given the importance of IDO2 in B cell responses, particularly in autoimmunity, mechanisms by which this protein can be specifically targeted are urgently needed. Much previous work has focused on the effect of the tryptophan analog 1MT in a variety of model systems. In some contexts, IDO pathway inhibition can alleviate disease (18, 19, 31) while in others, disease is exacerbated (22, 29, 34, 55) or has no effect at all (25, 33, 56, 57). Work with 1MT has also supported the idea that IDO may directly suppress antibody responses to vaccination (58). While these studies give us an important overview of the role of the IDO pathway, 1MT appears to act non-specifically. Given the potentially opposing roles of IDO1 and IDO2 as well as the potential non-enzymatic roles of both these proteins, methods for targeting IDO1 and IDO2 separately in B cells are strongly recommended.

In addition to 1MT, there are other, more specific inhibitors that have been described. The best studied small molecule inhibitor is epacadostat (Incyte), which selectively inhibits IDO1 without affecting IDO2 or TDO (59). Epacadostat has been extensively studied as a co-treatment with other immunotherapies in cancers but did not meet primary trial endpoints (60). It does, however, give us a potential tool to study inhibition of IDO1 and the role of IDO1 in B cells in systems where gene deletion is not a feasible option.

Antibodies against IDO1 or IDO2 also provide a mechanism for specific targeting of each enzyme. Our lab has developed an anti-IDO2 antibody that can successfully ameliorate arthritis in the KRN and CIA models (24). This antibody is able to access intracellular IDO2 *via* internalization mediated by the FcγRIIb receptor, and FcγRIIb on B cells is required *in vivo* for IDO2 Ig therapeutic activity, again supporting the importance of IDO2 in B cells in modulating autoimmune responses.

In addition to antibody therapy, our lab has also used a nanoparticle delivery system for anti-IDO2 siRNA (61). Like genetic knockouts or the anti-IDO2 antibody, targeting of IDO2 in B cells by this siRNA approach successfully reduces arthritis. This system is advantageous because it can be targeted to specific cell types (B cells in this case) and can be easily adapted to alter the siRNA being delivered to the target of interest.

## Discussion

In summary, several lines of evidence point to important roles for both IDO1 and IDO2 in B cell-mediated immune responses. In most models, IDO1’s function appears to be immunosuppressive for B cell activity, whereas IDO2 functions to positively mediate proinflammatory B cell responses (**Table 1**). Given their opposing immunomodulatory roles, it is therefore critical to distinguish the individual contribution of IDO1 vs. IDO2 for each system. Autoreactive B cell responses appear to be particularly impacted by IDO2, leading to various strategies to inhibit IDO2’s pathogenic function without affecting beneficial effects of IDO1. Although both IDO1 and IDO2 are tryptophan catabolizing enzymes, recent studies have identified functions independent of their enzymatic activity. As such, strategies that solely target enzymatic activity may not be effective inhibitors of IDO1/2 function mediating disease. Further work is needed to elucidate the upstream and downstream mediators of IDO1 and IDO2 function to identify pathways that can be targeted therapeutically.

**Table 1:** Effect of IDO1 and IDO2 on autoimmune and inflammatory disease.

Disease	Model(s)	IDO1 ko	IDO2 ko	Inhibitors
Arthritis	KRN	no effect (13, 14)	reduced (11, 13, 14)	reduced (18, 24, 61)
	CIA	Increased (22)	n.d.	reduced (24, 26) increased (22, 23) no effect (24, 25)
	JIA	no effect (20)	n.d.	n.d.
Psoriasis	IMQ	no effect (32)	increased (32)	n.d.
Multiple Sclerosis	EAE	increased (27, 28)	no effect (28)	increased (29, 30) reduced (31)
Lupus	Nba2	no effect (33)	n.d.	no effect (33)
	MRL/lpr	n.d.	n.d.	reduced (34) increased (19)
Allergy/Asthma	CHS	reduced (12)	reduced (11, 12)	n.d.
	Ag-induced asthma	reduced (37)	n.d.	n.d.
Immunization	T Independent Type I	increased (9) no effect (14)	no effect (14)	n.d.
	T Independent Type II	increased (9) no effect (14)	reduced (14)	n.d.
	T Dependent	no effect (9, 14)	no effect (14)	n.d.
Influenza	PR8	increased CD8 (38) no effect on Ab (14, 38)	reduced (14)	n.d.
Endotoxic Shock	LPS	reduced (39)	increased (40)	n.d.
Aspergillosis	*A. fumigatus*	reduced (41)	n.d.	n.d.

The inflammatory response in the listed models is indicated as increased, reduced,neither increased nor decreased (no effect), or not determined (n.d.).

## Author Contributions

All authors assisted with the writing of the manuscript and read and approved the submitted version.

## Funding

This work was supported by the Lankenau Medical Center Foundation and Main Line Health.

## Conflict of Interest

The authors declare that the research was conducted in the absence of any commercial or financial relationships that could be construed as a potential conflict of interest.

## Publisher’s Note

All claims expressed in this article are solely those of the authors and do not necessarily represent those of their affiliated organizations, or those of the publisher, the editors and the reviewers. Any product that may be evaluated in this article, or claim that may be made by its manufacturer, is not guaranteed or endorsed by the publisher.
